# A commentary on “Artificial intelligence-based prediction model for surgical site infection in metastatic spinal disease: a multicenter development and validation study”

**DOI:** 10.1097/JS9.0000000000003123

**Published:** 2025-08-06

**Authors:** Xiangge Liu

**Affiliations:** Department of Orthopedics, First Affiliated Hospital of Jinan University, Guangzhou, Guangdong Province, P. R. China

*Dear Editor*,

I read with great interest your recently published article, *“Artificial Intelligence-Based Prediction Model for Surgical Site Infection in Metastatic Spinal Disease: A Multicenter Development and Validation Study”*^[1]^. First of all, I would like to highly commend the authors for their tremendous efforts in conducting multicenter, prospectively collected data analyses and for developing and validating an AI model with excellent predictive performance. By targeting the long-standing clinical challenge of predicting surgical site infection (SSI) risk in patients with metastatic spinal disease, this study leverages advanced machine learning techniques to build a gradient boosting model and deploy a clinically usable AI application, offering a novel approach for individualized risk stratification and preventive strategy design. We have been assured that the article complies with TITAN Guidelines 2025-governing declaration and use of AI^[2]^.

However, upon reading the full text carefully – especially the *“Human vs. AI predictive performance comparison”* section – I believe there are certain methodological limitations in the design of the physician-versus-AI comparison experiment that warrant further discussion. In the methods, the authors clearly state that the five participating spine surgeons were experienced specialists at their respective centers (10–15 years of practice). Under blinded conditions, these surgeons independently estimated the probability of SSI for each patient using de-identified, structured data, including demographics, preoperative laboratory results, tumor history, and planned surgical parameters. Meanwhile, the AI model generated SSI risk probabilities based on the same set of features. On the surface, this appears to be a fair “same-input” comparison. However, the design in fact artificially restricts the range of information available to human experts.

In real-world clinical practice, surgeons do not assess SSI risk solely using limited, table-like structured variables. Instead, they routinely integrate richer, multi-layered, multimodal information – including complete imaging studies (beyond simple variables like “tumor type” or “number of segments”), imaging features such as tumor vascularity or patterns of bone destruction, detailed physical examination findings (e.g., wound condition, skin integrity, nutritional status), nuances in prior surgical history, the patient’s overall systemic status and functional reserve, and longitudinal medical records^[3]^. This contextual and multimodal information is indispensable in daily practice and forms the foundation of a surgeon’s clinical “experience” and “intuition”^[4]^. By enforcing a de-identified, tabular format with strict feature selection, the study design effectively deprives surgeons of the very context they rely on for holistic risk assessment.

A potential bias introduced by this design is that it does not truly compare the AI model and surgeons under realistic clinical conditions. Instead, it pits the AI’s optimal use of structured variables against human predictions handicapped by severely constrained input. While this design elegantly demonstrates the model’s advantage in handling standardized variables, it does not prove that AI can replace the comprehensive clinical judgment of experienced spine surgeons in practice. Furthermore, the paper does not provide evidence that the AI model can reproduce or surpass physician performance in real-world environments such as multidisciplinary case discussions, imaging review, or intraoperative decision-making.

Additionally, the authors report a striking difference in performance (AI AUC of 0.986 vs. surgeons’ AUCs of 0.572–0.627, P < 0.001). This pronounced gap likely reflects, at least in part, the imposed simplification and restriction of information available to clinicians. To more fairly evaluate the true clinical utility of AI, future studies might consider designs comparing “physician-alone” versus “physician + AI collaboration” conditions^[5,6]^, allowing surgeons to use their usual comprehensive assessment while optionally integrating AI risk predictions. Such designs could test whether AI support meaningfully improves risk detection and perioperative management, thus realizing AI’s potential as a true decision-support tool for enhancing care quality and safety.

Finally, I would like to again thank the authors for their forward-looking work in metastatic spinal disease and their contribution to exploring AI translation into clinical practice. I sincerely hope these comments and suggestions may offer useful perspectives for future research.

## Data Availability

None.
